# Enhanced pathogenicity and synergistic effects of co-infection with bovine viral diarrhea virus 1 and HoBi-like virus in cattle and guinea pigs

**DOI:** 10.3389/fvets.2024.1464745

**Published:** 2024-11-12

**Authors:** Hongliang Cui, Baoru Ren, Linglong Wang, Jian Chen, Jie Li, Wei Hu, Yang Yang

**Affiliations:** ^1^The State Key Laboratory of Reproductive Regulation and Breeding of Grassland Livestock, School of Life Sciences, Inner Mongolia University, Hohhot, China; ^2^Jinyu Biotechnology Co., Ltd., Hohhot, China

**Keywords:** bovine viral diarrhea virus 1, HoBi-like virus, co-infection, mortality, clinical signs, complete blood count

## Abstract

**Introduction:**

The Bovine Viral Diarrhea Virus 1 (BVDV1) and HoBi-like virus (BVDV3), both within the same genus, share genomic homology and exhibit low antigenic cross-reactivity despite presenting similar clinical manifestations. In 2021, a bovine respiratory disease complex (BRDC) outbreak on two cattle farms in the Inner Mongolia Autonomous Region of China resulted in ten fatalities.

**Methods:**

Metagenomic and metatranscriptomic analyses were used to identify viral agents, including a co-infection case. A genetic evolution analysis assessed the relationships with related strains. Experimental infections in guinea pigs and calves evaluated the pathogenicity of the viruses

**Results:**

Phylogenetic analysis of the BVDV3 isolate IM2201 revealed close relatedness to Brazilian strains, with 97.06% nucleotide homology to the highly virulent strain SV478/07. Experimental co-infection in guinea pigs resulted in more severe clinical signs, including fever, cough, diarrhea, and significant pathological changes, and led to a higher mortality rate (40%) compared to no mortality from single-virus infections with BVDV1 or BVDV3. Similarly, co-infected cattle exhibited more severe clinical signs, including bloody diarrhea and rectal temperatures exceeding 40°C, along with persistent viremia and nasal viral shedding from 7 to 21 days post-infection. Blood analysis revealed significant reductions in white blood cell counts, particularly in co-infected cattle.

**Discussion:**

This study highlights the enhanced pathogenicity and synergistic effects of BVDV1 and BVDV3 co-infection, exacerbating disease severity.

## Introduction

Bovine viral diarrhea (BVD) poses a significant threat to cattle health, manifesting in a variety of clinical conditions, including respiratory diseases, reproductive disorders, gastroenteritis, mucosal disease (MD), and persistent infections (1, 2). BVD is caused by the bovine viral diarrhea virus (BVDV), a member of the genus *Pestivirus* within the family *Flaviviridae* (3). The International Committee on Taxonomy of Viruses (ICTV) classifies BVDV into three genotypes: BVDV1 (*Pestivirus* A), BVDV2 (*Pestivirus* B), and BVDV3 (HoBi-like *Pestivirus* or *Pestivirus* H). BVDV1 is further subdivided into at least 23 subtypes (1a–1w), BVDV2 into 5 subtypes (2a–2e), while BVDV3 has been identified with at least four subtypes (4, 5). Initially discovered in fetal bovine serum (FBS) from Brazil, BVDV3 has been observed in cattle across Asia, Europe, and South America and is associated with clinical presentations similar to those caused by BVDV1 or BVDV2 in cattle and small ruminants (6, 7).

BVDV is classified into cytopathic (CP) and noncytopathic (NCP) biotypes based on their ability to induce cytopathic effects (CPE) in host cells. Typically, CP BVDV induces apoptosis, whereas NCP BVDV inhibits apoptosis, leading to immune dysfunction (8, 9). However, some virulent NCP BVDV strains have demonstrated the ability to induce apoptosis *in vitro* (10). Although both biotypes are pathogenic, only NCP strains result in persistent infection (PI) in cattle, posing significant challenges for BVDV prevention and eradication due to continuous high-level virus shedding and horizontal transmission within herds. Efforts to control BVDV focus on maximizing immunity and minimizing herd exposure to the virus through vaccination, biosecurity measures, and identification of reservoirs. In North America, a multidimensional approach involving vaccination, biosecurity, and reservoir identification is implemented to control BVD (11). In Europe, several countries and regions have implemented mandatory or voluntary BVDV control and/or eradication programs, tailored to country-specific factors (12). These programs vary by region, considering factors such as BVDV incidence, animal population density, movement, contact with wildlife, producer compliance, circulating BVDV strains, industry type, and institutional support (13). Globally, BVDV results in substantial economic losses due to acute infections, induced immunosuppression, and resource-intensive control measures, including vaccination, testing, and culling.

According to the Chinese National Bureau of Statistics, China had 102.16 million head of cattle as of 2022 (14). In 2023, the Inner Mongolia Autonomous Region had a cattle inventory of 9.477 million, ranking first among all provinces in China (15). A systematic review and meta-analysis of 27,530 cows in China revealed a BVDV seroprevalence of 57.0% (16). To date, no official BVDV control and eradication program has been implemented in China, and only commercial inactivated BVDV1 vaccines are available (17). Reports suggest that these vaccines may not effectively prevent or control BVDV3, complicating BVD epidemiology in China and potentially leading to economic losses in countries where animal husbandry is a major industry. Emerging BVDV biotypes may exhibit greater antigenic differences or induce more severe lesions than previous strains (18).

The objective of this study was to investigate the pathogenesis and virulence of BVDV1 and BVDV3 isolates from China in cattle. This investigation involved both natural infections and experimental co-infections of BVDV1 and BVDV3 in cattle and guinea pigs, aiming to determine whether co-infection leads to acute fatal infections, respiratory disease, or significant changes in white blood cell, lymphocyte, and neutrophil counts.

## Results

### Clinical detection and metagenomic analysis of bovine viral respiratory pathogens in cattle outbreaks

**Figure 1:**
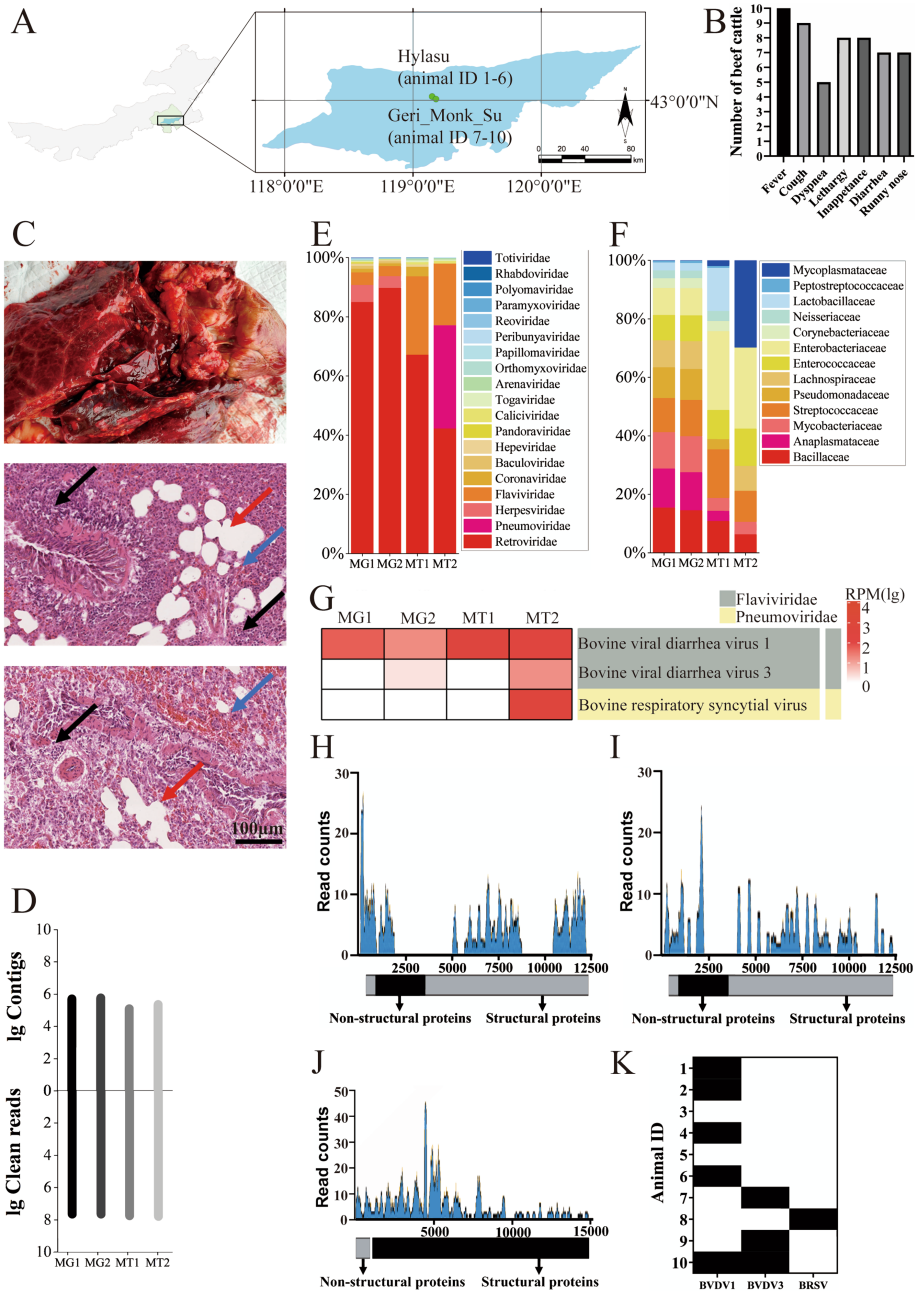
Identification of viruses in BRDC-related dead cattle lung samples in Inner Mongolia. (A) Geographical location of BRDC-affected lung samples in the Inner Mongolia Autonomous Region, China. The top point denotes the Hylasu cattle farm (43.03°N, 119.15°E), where six samples (animal IDs 1–6) were collected. The bottom point denotes the Geri Monk Su farm (43.01°N, 119.18°E), where four samples (animal IDs 7–10) were collected. (B) Clinical signs observed in BRDC-affected cattle prior to death. The *x*-axis shows the clinical signs, and the *y*-axis represents the number of cattle exhibiting each clinical signs. (C) Histopathological analysis of BRDC-affected lungs from deceased cattle. Blue arrows indicate capillary congestion in alveolar walls, black arrows denote peripheral lymphocytic and monocytic infiltrates, and red arrows indicate emphysema. (D) Overview of read counts and contig sequences from bovine lung samples. MG1 and MG2 correspond to metagenomic sequencing, while MT1 and MT2 correspond to the metatranscriptomic sequencing. (E) Proportion of reads associated with viral families among all viral reads. Viral families are shown on the right. (F) Proportion of reads associated with bacterial families among all bacterial reads. Bacterial families are shown on the right. (G) Heatmap illustrating BRDC-related viral contigs in each sample, displayed using log10-transformed read values. (H–J) Viral genome coverage and sequencing depth analysis for BVDV1, BVDV3, and BRSV. (K) Detection of BRDC-associated viral RNA in the lung tissue of 10 deceased cattle using RT-PCR. Black denotes positive results, while white denotes negative results.

Ten cattle with fatal bovine respiratory disease complex (BRDC) were identified between June and September 2021 on two cattle farms in the Inner Mongolia Autonomous Region, China (Figure 1A). The recorded clinical signs, listed from most to least common, included: fever (*n* = 10, 100%), cough (*n* = 9, 90%), lethargy (*n* = 8, 80%), inappetance (*n* = 8, 80%), diarrhea (*n* = 7, 70%), runny nose (*n* = 7, 70%), and dyspnea (*n* = 5, 50%) (Figure 1B). The animal with ID 10 exhibited particularly severe clinical signs before death. Lung samples were collected for histopathological analysis, revealing significant pathological changes, including congestion of capillaries in the alveolar walls, peripheral lymphocytic and monocytic infiltrates, and emphysema (Figure 1C).

To identify the causative agents of the fatal disease, lung samples from deceased cattle on the farm were pooled for metagenomic and metatranscriptomic analyses. Metagenomic and metatranscriptomic sequencing resulted in 43 to 60 million reads per pool (205,091,743 reads in total), which were assembled *de novo* into 125,627 to 582,782 contigs (Figure 1D). Among virus-related sequences, *Retroviridae* accounted for about 75%, *Flaviviridae* accounted for about 11%, and *Pneumoviridae* accounted for about 6%. Considering the possibility of contamination in *Retroviridae*, it was found that the virus family with the largest proportion was BRDC-related virus family (Figure 1E). Additionally, in the bacterial-related sequences, no bacterial families related to BRDC was found (Figure 1F).

Annotation results indicated that the most abundant viruses belonged to the families *Flaviviridae* and *Pneumoviridae* (Figure 1G). A 6,908-base contig of the BVDV1 genome was assembled with 54.9% coverage, identifying ORFs encoding Npro, C, Erns, NS2, NS3, NS4A, NS4B, NS5A, and NS5B (Figure 1H). A 6,134-base contig of the BVDV3 genome was assembled with 50.7% coverage, identifying ORFs encoding Npro, C, Erns, E1, NS3, NS4A, NS4B, NS5A, and NS5B (Figure 1I). An 11,672-base contig of the bovine respiratory syncytial virus (BRSV) genome was assembled with 77.2% coverage, identifying ORFs encoding all proteins (Figure 1J). To further confirm viral RNA in the cattle, reverse transcription-PCR (RT-PCR) assays were conducted to detect BVDV1, BVDV3, and BRSV. Five cattle tested positive for BVDV1, three for BVDV3, and one for BRSV (Figure 1K). Both BVDV1 and BVDV3 were detected in the animal with ID 10, indicating a suspected co-infection that may have contributed to the severe clinical signs observed.

### Isolation and phylogenetic analysis of BVDV1 and BVDV3 from fatal cattle cases

**Figure 2:**
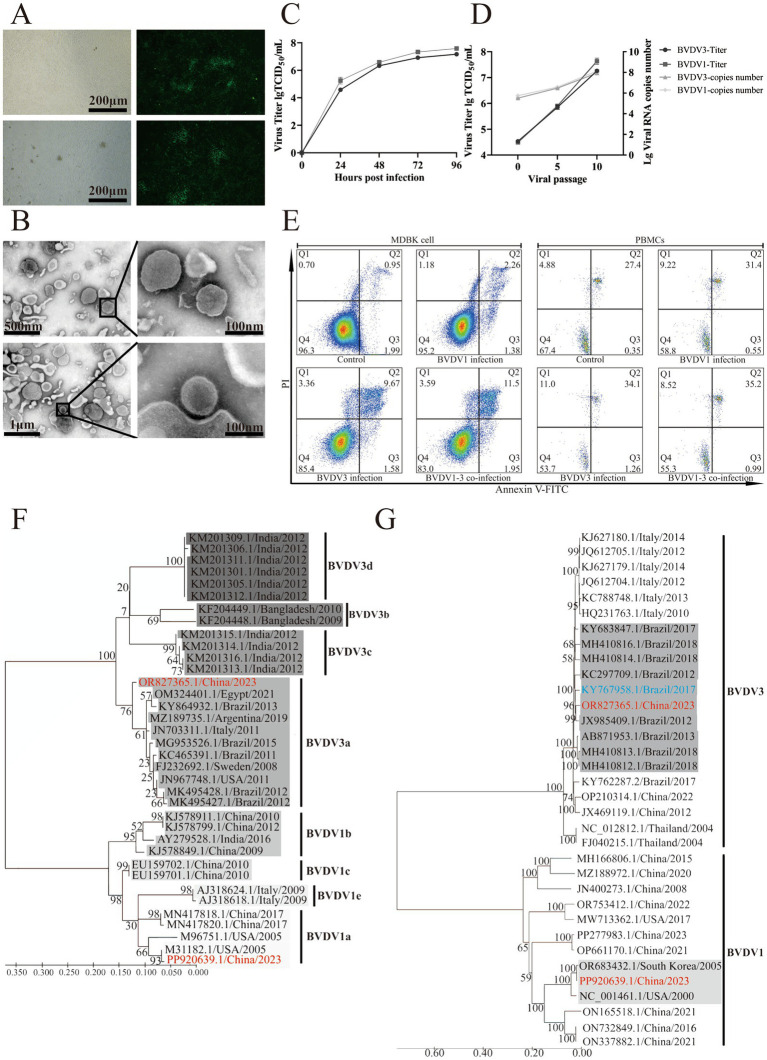
Morphological and phylogenetic analysis of BVDV1 and BVDV3. (A) BVDV1 (top) and BVDV3 (bottom) cultured in MDBK cells were identified using indirect IF. (B) Photographs of BVDV1 (top) and BVDV3 (bottom) viral particles were observed via electron microscopy. (C) The viral titers and RNA copy numbers of BVDV1 and BVDV3 at passages 0, 5, and 10 in MDBK cells were quantified. (D) Growth curves of BVDV1 and BVDV3 in MDBK cells. BVDV1 and BVDV3 showed similar proliferation trends. Each data point represented the average of three replicates. The error bar indicates standard deviation. (E) Forty-eight hours post-infection of MDBK cells and PBMCs with BVDV1 and/or BVDV3, apoptosis (%) was quantified using flow cytometry. (F) Maximum likelihood phylogenetic trees of BVDV1 and BVDV3 isolated in this study, based on 5′UTR sequences, were constructed. Red indicates the strain isolated in this study, while background color shading denotes subtype classification. Trees are mid-point rooted and scaled according to the number of amino acid or nucleotide substitutions per site, with bootstrap values displayed at key nodes. (G) Maximum likelihood phylogenetic trees of BVDV1 and BVDV3 isolated in this study, based on full-length sequences, were constructed. Red indicates the strain isolated in this study, blue indicates a more virulent strain, and gray indicates the strain isolated in this study on the same branch. The trees are mid-point rooted and scaled according to the number of amino acid or nucleotide substitutions per site, with bootstrap values displayed at key nodes.

A BVDV1 strain, designated IM2202, was isolated from cattle with animal ID 1, while a BVDV3 strain, designated IM2201, was isolated from cattle with animal ID 9, both identified as single infections. Following 10 passages on MDBK cells and PBMCs at a 1:10 ratio, no significant cytopathic effects were observed in BVDV1-and BVDV3-infected cells (Figure 2A). Purified viruses were visualized using electron microscopy, revealing diameters of approximately 100 nm for both BVDV1 and BVDV3 (Figure 2B). BVDV1 and BVDV3 were tested via indirect immunofluorescence assay (IFA) using specific antibodies for each virus. At passage 5 on MDBK cells and PBMCs, the viral titers of BVDV1 and BVDV3 were below 10^6^ TCID_50_, and the viral RNA copy numbers were below 5 × 10^6^. By passage 10, the viral TCID_50_ of BVDV1 and BVDV3 reached 10^7^, with viral RNA copy numbers reaching 8 × 10^7^ (Figure 2C). The growth curves showed that the viral load in the cell supernatant increased rapidly 24–48 h post-infection. The viral load in the cell supernatant leveled off 72–96 h post-infection. The results indicated that the replication kinetics between BVDV1 and BVDV3 were similar (Figure 2D).

To investigate the apoptosis induced by different BVDV strains in MDBK cells and bovine PBMCs, the proportion of apoptotic cells in mock-infected MDBK cells and PBMCs was found to be 2.94 and 27.75%, respectively, as shown in Figure 2E. The proportions of apoptotic cells in BVDV1-infected, BVDV3-infected, and co-infected MDBK cells were 3.64, 11.25, and 13.45%, respectively, while in PBMCs, these proportions were 31.95, 35.36, and 36.19%. These results suggest that co-infection with BVDV1 and BVDV3 induces enhanced host cell apoptosis (Figure 2E).

Phylogenetic analysis of the 5′ UTR indicates that IM2201 belongs to genotype 3a, which is prevalent in Brazil, while IM2202 belongs to genotype 1a, prevalent in the USA (Figure 2F). The full-length phylogenetic tree, based on complete genome sequences, shows that IM2201 clusters with Brazilian strains, including CH-KaHo/cont, SV478/07, and LVRI/cont-1, with nucleotide homology of 97.06, 96.34, and 95.88%, respectively. Similarly, the phylogenetic tree based on the complete genome sequences of BVDV1 indicates that strain IM2202 clusters with strains KD26 and NADL, showing nucleotide homology of 99.75 and 96.54%, respectively (Figure 2G).

### Pathological and immunological characteristics of BVDV1 and/or BVDV3 infection in guinea pigs

**Figure 3:**
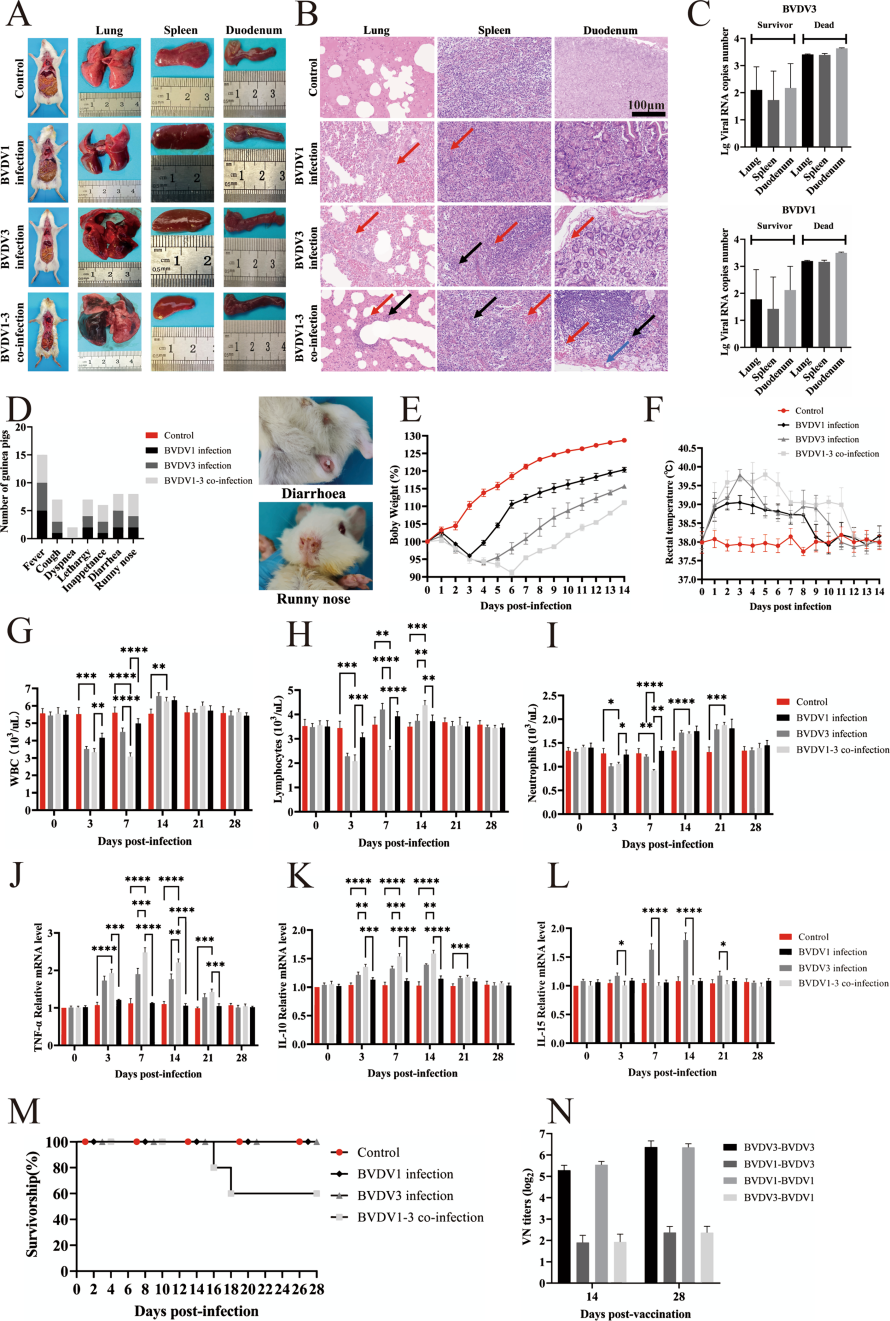
Pathological and clinical signs of BVDV1 and/or BVDV3 infection in guinea pigs. (A) Dissections of whole bodies and organs of guinea pigs from control, BVDV1-infected, BVDV3-infected, and BVDV1 and BVDV3 co-infected groups. (B) HE staining of lung, spleen, and duodenum lesions in guinea pigs at 28 dpi. Red arrows indicate hemorrhage, black arrows indicate peripheral lymphocytic and monocytic infiltrates, and blue arrows indicate intestinal gland necrosis. (C) Viral genomic RNA copy numbers of BVDV3 (top) and BVDV1 (bottom) in the lungs, spleen, and duodenum of deceased and surviving guinea pigs. (D) Clinical signs in guinea pigs induced by BVDV1, BVDV3, or co-infection from 0 dpi to 28 dpi. (E) Percentage changes in body weight of guinea pigs post-infection from 0 dpi to 14 dpi. (F) Rectal temperature changes in guinea pigs post-infection from 0 dpi to 14 dpi. (G) WBC counts in guinea pigs infected with different BVDV strains from 0 dpi to 28 dpi. Standard error bars are shown, with asterisks indicating statistically significant differences. (H) Lymphocyte counts in guinea pigs infected with different BVDV strains from 0 dpi to 28 dpi. Standard error bars are shown, with asterisks indicating statistically significant differences. (I) Neutrophil counts in guinea pigs infected with different BVDV strains from 0 dpi to 28 dpi. Standard error bars are shown, with asterisks indicating statistically significant differences. (J) TNF-α expression levels in spleen tissue of infected guinea pigs relative to the control group. Data are expressed as fold difference of mRNA expression normalized to the housekeeping gene (GAPDH), relative to the values obtained for uninfected guinea pigs. (K) IL-10 expression levels in spleen tissue of infected guinea pigs relative to the control group. Data are expressed as fold difference of mRNA expression normalized to the housekeeping gene (GAPDH), relative to the values obtained for uninfected guinea pigs. (L) IL-15 expression levels in spleen tissue of infected guinea pigs relative to the control group. Data are expressed as fold difference of mRNA expression normalized to the housekeeping gene (GAPDH), relative to the values obtained for uninfected guinea pigs. (M) Survival rates of guinea pigs inoculated with various BVDV strains. (N) Antigenic cross-reactivity to BVDV1 and BVDV3 measured at 14 and 28 days post-infection. Antibody responses are presented as median VN titers.

Guinea pigs were intranasally inoculated with 100 μL of 10^5^ TCID_50_ of passage 5 BVDV1 and/or BVDV3. Twenty-eight days post-infection, the guinea pigs were euthanized for autopsy, during which lung, spleen, and duodenum tissues were collected for pathological examination and viral titer assessment. No pathological changes were observed in the mock-infected guinea pigs, whereas hemorrhage and necrosis were evident in the BVDV-infected group (Figure 3A). More pronounced pathological changes associated with BVDV were observed in the guinea pigs co-infected with BVDV1 and BVDV3. The lungs and spleens of the co-infected guinea pigs exhibited peripheral lymphocytic and monocytic infiltrates along with hemorrhage. In the duodenum of the co-infected guinea pigs, necrosis of intestinal glands, extensive lymphocyte infiltration, and hemorrhage were observed, characteristic of MD. Similar pathological changes were observed in other groups, though none were as severe as those in the BVDV1 and BVDV3 co-infected guinea pigs (Figure 3B). The BVDV copy numbers in the lungs, spleens, and duodena of deceased co-infected guinea pigs exceeded 1,300 copies/μg, yet no live virus was detected in these tissues. In all surviving guinea pigs, the viral load in the organs remained below 900 copies/μg at 28 days post-infection, with no significant difference between the BVDV1 and BVDV3 viral loads (Figure 3C). The BVDV1 and BVDV3 co-infected guinea pigs exhibited more severe clinical signs compared to those infected with either virus alone. The clinical signs observed in the co-infected guinea pigs, listed in order of prevalence, included: fever (*n* = 5, 100%), cough (*n* = 4, 80%), runny nose (*n* = 4, 80%), lethargy (*n* = 3, 60%), inappetance (*n* = 3, 60%), diarrhea (*n* = 3, 60%), and dyspnea (*n* = 2, 40%) (Figure 3D). The body weight of the BVDV1-infected, BVDV3-infected, and co-infected guinea pigs decreased to below 96, 94, and 91%, respectively, after 3 dpi (Figure 3E). The rectal temperature of the co-infected guinea pigs increased to a peak of over 40.29°C, which was 1°C higher than that of the BVDV1-infected guinea pigs (Figure 3F).

The average white blood cell (WBC) count in mock-infected guinea pigs was approximately 5.5 × 10^3^/μL, while in BVDV1-infected, BVDV3-infected, and co-infected guinea pigs, the WBC counts decreased to 4.2, 3.5, and 3.1 × 10^3^/μL, respectively. The WBC counts subsequently increased to above 6.3, 6.6, and 6.3 × 10^3^/μL at 7–14 dpi, returning to normal levels at 21–28 dpi (Figure 3G). In mock-infected guinea pigs, the average lymphocyte count was approximately 3.5 × 10^3^/μL. In BVDV1-infected, BVDV3-infected, and co-infected guinea pigs, the lymphocyte counts decreased to 3.1, 2.3, and 2.1 × 10^3^/μL at 3–7 dpi, followed by an increase to over 3.9, 4.2, and 4.4 × 10^3^/μL at 7–14 dpi, and returning to normal by 21–28 dpi (Figure 3H). Similarly, the average neutrophil count in mock-infected guinea pigs was approximately 1.328 × 10^3^/μL. In BVDV1-infected, BVDV3-infected, and co-infected guinea pigs, neutrophil counts dropped to 1.255, 1.006, and 0.918 × 10^3^/μL at 3–7 dpi, then increased to over 1.806, 1.785, and 1.877 × 10^3^/μL at 14–21 dpi, and returned to normal levels by 21–28 dpi (Figure 3I). At 3–14 dpi, the BVDV1 and BVDV3 co-infected guinea pigs exhibited significant differences in WBC and lymphocyte counts compared to the other groups, while neutrophil counts showed significant differences at 3–21 dpi. These findings indicate that co-infection with BVDV1 and BVDV3 induces more severe alterations in total and differential leukocyte counts in guinea pigs.

The average TNF-α levels in the BVDV3-infected and BVDV1 and BVDV3 co-infected guinea pigs were significantly higher than those in the mock-infected group from 3 dpi to 14 dpi, returning to normal at 21–28 dpi. From 3 to 21 dpi, the BVDV1 and BVDV3 co-infected guinea pigs exhibited significant differences compared to the other groups (Figure 3J). The average IL-10 level in the BVDV1 and BVDV3 co-infected guinea pigs was significantly higher than those in the mock-infected group from 3 dpi to 14 dpi, returning to normal at 21–28 dpi (Figure 3K). IL-15 is an essential cytokine for NK cell proliferation during the innate immune response against viruses (19). From 3 dpi to 14 dpi, only the average IL-15 level in the BVDV3-infected guinea pigs was significantly higher than that in the other groups, returning to normal at 21–28 dpi (Figure 3L). Therefore, the results suggest that BVDV1 inhibits the transcription of IL-15, which may serve as a potential immunosuppressive mechanism.

Two of the guinea pigs inoculated with BVDV1 and BVDV3 exhibited severe BVDV-related clinical signs and succumbed at 16–18 dpi, resulting in a total mortality rate of 40% (Figure 3M). The virus neutralization (VN) titers of guinea pigs infected with BVDV1 or BVDV3 against their respective viruses exceeded 1:64 at 14 dpi. In contrast, the VN titers of BVDV1-infected guinea pigs against BVDV3, as well as those of BVDV3-infected guinea pigs against BVDV1, were approximately 1:4, indicating limited cross-neutralization (Figure 3N). Viral RNA was identified in nasal swabs (NS) and whole blood (WB) collected at two-day intervals post-infection (Table 1). Viremia was consistently observed in the BVDV1 and BVDV3 co-infected guinea pigs, beginning at 3 dpi and persisting until 21–28 dpi, with 100% of the guinea pigs exhibiting viremia. In contrast, viral RNA was detected in 40% of guinea pigs infected with BVDV1 and 60% of those infected with BVDV3. Additionally, viral RNA in NS was identified across all three infection groups between 7 and 14 dpi. Specifically, viral RNA in NS was identified in 40% of guinea pigs singly infected with BVDV1 or BVDV3, and in 100% of those co-infected with both BVDV1 and BVDV3.

**Table 1:** Viral RNA in NS and WB of guinea pigs was detected by RT-PCR.

Strain	Animal ID	Days post-infection									
		Day 3		Day 7		Day 14		Day 21		Day 28	
		NS	WB	NS	WB	NS	WB	NS	WB	NS	WB
IM2202	1	−	−	−	1+	1+	1+	−	−	−	−
	2	−	−	−	−	−	−	−	−	−	−
	3	−	−	1+	−	1+	1+	−	1+	−	−
	4	−	−	−	−	−	−	−	−	−	−
	5	−	−	−	−	−	−	−	−	−	−
IM2201	1	−	−	−	−	−	−	−	−	−	−
	2	−	−	3+	3+	3+	−	−	−	−	−
	3	−	−	−	−	3+	3+	3+	−	−	−
	4	−	−	−	−	−	3+	−	3+	−	−
	5	−	−	−	−	−	−	−	−	−	−
IM2202-IM2201	1	−	−	−	3+	1+/3+	1+/3+	−	3+	−	−
	2	−	−	−	1+	1+	1+/3+	−	−	−	−
	3	−	3+	1+/3+	3+	1+/3+	1+/3+	3+	3+	−	−
	4	−	−	1+/3+	−	1+/3+	3+	−	−	−	−
	5	−	−	−	−	1+/3+	−	−	−	−	−

“1+” represents BVDV1 positive, “3+” represents BVDV3 positive, and “−” represents negative.

### Clinical manifestations of BVDV1 and/or BVDV3 infection in calves

**Figure 4:**
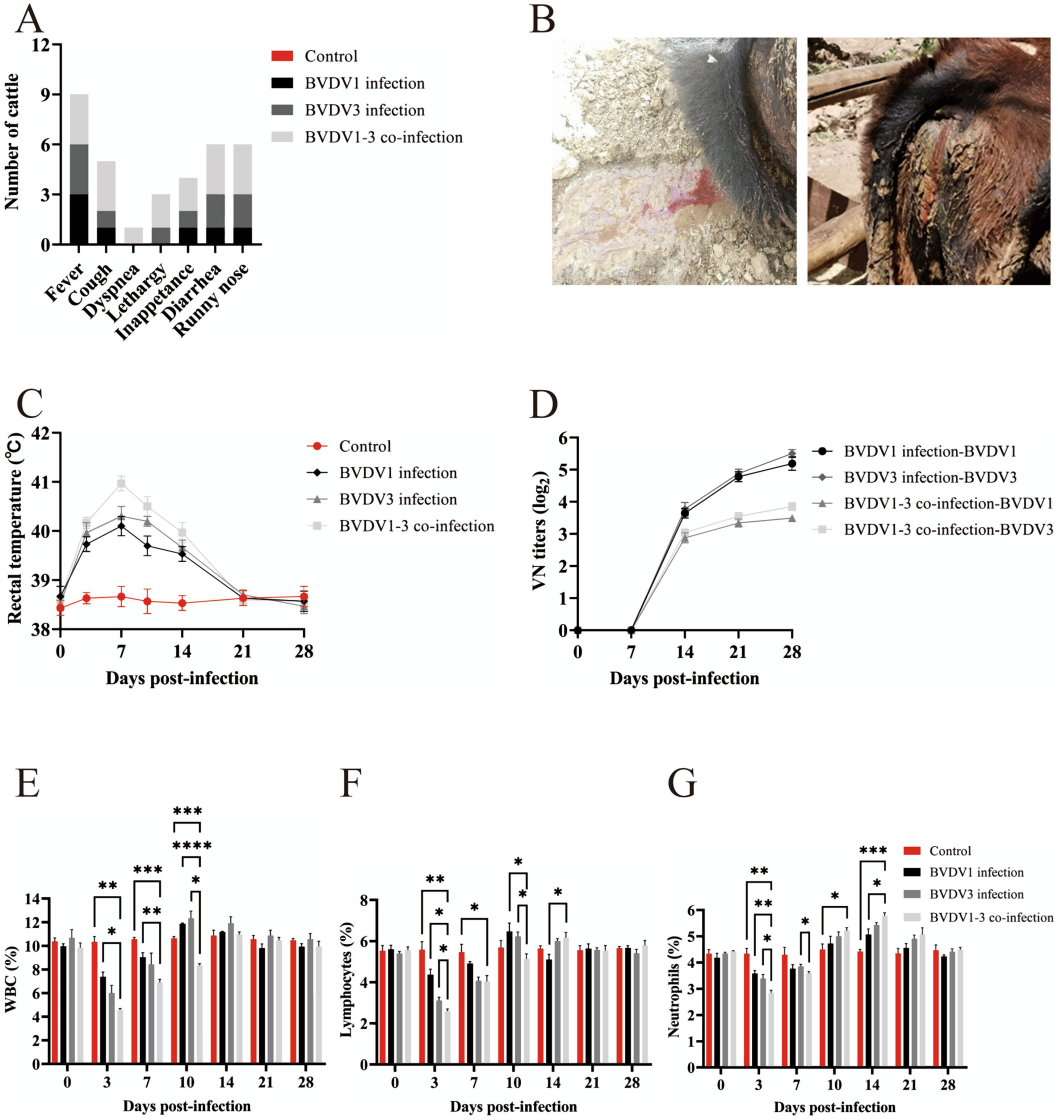
Clinical signs in calves infected with BVDV1 and/or BVDV3. (A) Clinical signs in calves following BVDV1, BVDV3, or co-infection with BVDV1 and BVDV3 from 0 dpi to 28 dpi. (B) Photographs depicting calves with diarrhea and bloody stools. (C) Rectal temperature variations in calves post-infection from 0 dpi to 28 dpi. (D) Antibody responses shown as median VN titers. (E–G) WBC, lymphocyte, and neutrophil counts in BVDV-infected calves from 0 dpi to 28 dpi. Standard error bars are indicated, with asterisks marking significant differences. Only differences between the BVDV1 and BVDV3 co-infection group and other groups are shown.

Calves were nasally inoculated with 3 mL of 3 × 10^6^ TCID_50_ of passage 5 BVDV1 and/or BVDV3 and subsequently observed for 28 days. More severe clinical signs were observed in calves co-infected with BVDV1 and BVDV3 compared to those singly infected with either virus (Figure 4A). At 7 dpi, two calves co-infected with BVDV1 and BVDV3 exhibited severe bloody diarrhea (Figure 4B). Rectal temperatures in infected calves exceeded 40°C and normalized between 21–28 dpi (Figure 4C). *Pestivirus* antibodies emerged at 10 dpi in infected calves, peaking at 21–28 dpi with a maximum VN titer of 1:64 (Figure 4D). The average WBC count in mock-infected calves was approximately 10.2 × 10^3^/μL, while in BVDV1-infected, BVDV3-infected, and co-infected calves, the count dropped below 7.4, 6.0, and 4.6 × 10^3^/μL, respectively, at 3 dpi. The count increased to over 11.9, 12.3, and 11.0 × 10^3^/μL at 10–14 dpi, and returned to normal between 21 and 28 dpi (Figure 4E). The average lymphocyte count in mock-infected calves was about 5.5 × 10^3^/μL. The lymphocyte count in BVDV1-infected, BVDV3-infected, and co-infected calves dropped below 4.4, 3.1, and 2.6 × 10^3^/μL, respectively, at 3 dpi, then increased to over 6.5, 6.2, and 6.2 × 10^3^/μL from 10 to 14 dpi, returning to normal between 21 and 28 dpi (Figure 4F). Similarly, the average neutrophil count in mock-infected calves was approximately 4.32 × 10^3^/μL. The neutrophil count in BVDV1-infected, BVDV3-infected, and co-infected calves dropped below 3.58, 3.39, and 2.84 × 10^3^/μL, respectively, at 3 dpi, increasing to over 5.07, 5.44, and 5.78 × 10^3^/μL at 10–14 dpi, returning to normal between 21 and 28 dpi (Figure 4G). At 3–14 dpi, co-infected calves exhibited significant differences in lymphocytes and neutrophils compared to other groups, while WBC counts showed significant differences at 3–10 dpi. Viral RNA was detected in NS and WB collected from all calves (Table 2). Viremia and nasal viral shedding were observed in BVDV1 and BVDV3 co-infected calves from 7 dpi to 21 dpi, with all calves testing positive for viral RNA. In contrast, 33.3% of BVDV1-infected calves and 66.6% of BVDV3-infected calves tested positive for viral RNA. This indicates that co-infection with BVDV1 and BVDV3 in calves results in more severe clinical signs, akin to those seen in guinea pigs.

**Table 2:** Viral RNA in NS and WB of calves was detected by RT-PCR.

Strain	Animal ID	Days post-infection											
		Day 3		Day 7		Day 10		Day 14		Day 21		Day 28	
		NS	WB	NS	WB	NS	WB	NS	WB	NS	WB	NS	WB
IM2202	1	−	−	−	1+	1+	1+	1+	−	1+	−	−	−
	2	−	−	−	−	−	−	−	−	−	−	−	−
	3	−	−	−	−	−	−	−	−	−	−	−	−
IM2201	1	−	−	3+	−	3+	−	3+	−	−	−	−	−
	2	−	−	3+	3+	3+	3+	3+	−	3+	−	−	−
	3	−	−	−	−	−	−	−	−	−	−	−	−
IM2201-IM2202	1	−	−	1+/3+	1+/3+	1+/3+	1+/3+	3+	3+	3+	−	−	−
	2	−	−	1+	1+/3+	1+/3+	1+/3+	1+	−	1+	−	−	−
	3	−	−	1+/3+	1+/3+	1+/3+	1+/3+	1+/3+	3+	−	−	−	−

“1+” represents BVDV1 positive, “3+” represents BVDV3 positive, and “−” represents negative.

## Materials and methods

### Sample collection

Six lung samples from dead cattle (animal ID 1–6) were collected from the Hylasu cattle farm (43.03°N, 119.15°E), while four samples (animal ID 7–10) were obtained from Geri Monk Su farm (43.01°N, 119.18°E) in the Inner Mongolia Autonomous Region, China, between June and September 2021. All cattle presented with severe clinical signs of BRDC and had not been vaccinated against BVDV. Samples were collected within 6 h after death from regions with pronounced lung lesions. Each sample was divided into two portions: one was stored in a sterile container at −80°C, and the other was immediately fixed in a 4% paraformaldehyde solution for histopathological examination.

### DNA/RNA extraction, library preparation, and sequencing

Lung samples from each cattle farm were pooled, and DNA was extracted using the DNeasy Blood & Tissue Kit (Qiagen, Germany). RNA was extracted using the QIAamp DSP Viral RNA Mini Kit (Qiagen, Germany), following the manufacturer’s instructions. DNA libraries were constructed using the NEBNext Ultra DNA Library Prep Kit for Illumina (NEB, United States). rRNA-depleted messenger RNA was used for sequencing through the construction of complementary DNA libraries using the NEBNext^®^ Ultra^™^ II Directional RNA Library Prep Kit for Illumina^®^ (NEB, United States). Library quality was assessed using an Agilent Bioanalyzer 2100. Sequencing was performed on the Illumina PE150 platform (Beijing Nuohe, China). Raw reads were filtered to remove adapter sequences and low-quality sequences using fastp (v0.19.4), and reads mapped to the host genomic DNA using BWA MEM (v0.7.17-r1188) were excluded to obtain clean data. Contigs were generated through *de novo* assembly of the clean data using MEGAHIT (v1.1.3) and Trinity (v2.9.0), with a minimum assembly length of 300 bp. The sequence data from each library were assembled separately.

### Viral identification and confirmation

The Non-Redundant Protein Sequence Database (NR) and Nucleotide Sequence Database (NT) from NCBI were utilized for viral identification. DIAMOND (v2.1.8) and BLASTN (v2.13.0, https://blast.ncbi.nlm.nih.gov/) were employed to construct a sub-library of viral proteins and nucleic acids for preliminary alignment and annotation (20). Contigs were employed for subsequent alignment and annotation. The obtained annotation data were utilized to create a columnar stacking diagram using Origin 2021 software (OriginLab Corp.). Subsequently, reads of pathogens associated with BRDC were visualized through a heatmap generated using the complexheatmap (2.13.1) package in R (21). The “Map to Reference” function in Geneious Prime (v2021.0.3, https://www.geneious.com/) was employed to align the clean reads from the four libraries to the viral genome, facilitating the analysis of coverage for each target virus and the corresponding read abundance. PCR primers for detecting bovine respiratory pathogens (BVDV1, BVDV3, and BRSV) were synthesized by Shanghai Shenggong Biotechnology Service Co., Ltd. (Supplementary Table S1).

### Cells, viruses, and antibodies

Whole blood was collected from BVDV-negative calves, and peripheral blood mononuclear cells (PBMCs) were isolated using Ficoll Histopaque solution (Sigma-Aldrich). Madin-Darby bovine kidney (MDBK) cells were cultured in Dulbecco’s modified Eagle’s medium (DMEM) supplemented with 10% fetal bovine serum (FBS) at 37°C in a 5% CO₂ incubator. BVDV1a (IM2202, GenBank accession number PP920639.1) and BVDV3a (IM2201, GenBank accession number OR827365.1) were isolated from deceased cattle. Monoclonal antibodies against BVDV1 were procured from Veterinary Medical Research & Development (VMRD, United States). The IM2201-E2 protein (GenBank accession number WPM94674.1) was expressed in HEK293 cells. Rabbits were administered a subcutaneous injection of E2 protein (500 μg), mixed with complete Freund’s adjuvant (Sigma-Aldrich; Merck KGaA) in a 1:1 ratio. After 2 weeks, the rabbits received three booster injections of E2 protein (500 μg) mixed with incomplete Freund’s adjuvant (1:1) at two-week intervals. Polyclonal antibodies against the E2 protein were purified from the rabbit immune sera via ammonium sulfate precipitation, followed by purification with the Serum Antibody Purification Kit (Abcam, United States).

### Viral sequencing and phylogenetic analysis

Total RNA was extracted from BVDV-infected MDBK cells using the RNAiso Plus reagent (Takara Bio, Japan). Reverse transcription reactions were performed using the PrimeScript^™^ RT Reagent Kit with gDNA Eraser (Takara Bio, Japan). Nine and twelve primer pairs were designed to amplify the complete genomes of BVDV1 and BVDV3, respectively (Supplementary Table S2). The PCR products were cloned into the pMD-19T vector (Takara Bio, Japan) and sequenced by Sangon BioEngineering Co., Ltd. (China). Full-length viral sequences were aligned with published reference strains in GenBank using MEGA 10.0 software. The maximum likelihood method with the TN93 model was employed to construct the phylogenetic tree. The bootstrap method with 1,000 bootstrap tests was used to improve the reliability of the results.

### Immunofluorescence and TCID_50_ assay

After 2 days of infection, the supernatant was discarded, and the MDBK cells were fixed with 4% paraformaldehyde for 20 min at room temperature, followed by blocking with 5% bovine serum albumin (BSA) for 1 h at room temperature. The cells were incubated at 4°C for 16 h with primary antibodies against BVDV1 or BVDV3, then washed with PBS and incubated with secondary antibodies (Goat Anti-Mouse/Rabbit IgG H&L, Abcam, United Kingdom) for 1 h at 37°C in the dark. Finally, the cells were stained with DAPI for 15 min at room temperature and observed using a fluorescence microscope (TS100, Nikon). The images were analyzed using Image-Pro Plus 6.0 software. The 50% tissue culture infective dose (TCID₅₀) was determined using the Reed-Muench method.

### Growth curves of BVDV

MDBK cells were plated into 6-well cell culture plates and cultured in DMEM containing 10% FBS. After 12 h, MDBK cells were inoculated with 0.1 MOI of BVDV1 and BVDV3. Cell supernatants were collected at 24 h, 48 h, 72 h, and 96 h post-infection, and three biological replicates were set for each time point. The TCID_50_ of the virus at each time point was measured by IF.

### Viral purification and electron microscopy

MDBK cells infected with BVDV1 or BVDV3 for 96 h were centrifuged at 600 × g for 20 min at 4°C to precipitate cell debris. The supernatant was incubated with PEG6000 and NaCl for 16 h at 4°C, followed by centrifugation at 10,000 × g for 1 h to obtain the viral pellet. The precipitate was resuspended in PBS and transferred to a sucrose gradient ultracentrifugation tube. The tube was ultracentrifuged at 100,000 × g for 2 h at 4°C using an SW32Ti rotor (Optima XPN-100, Beckman, United States). The viral layer was extracted, transferred to a PBS-containing tube, and centrifuged at 100,000 × g for 1 h to remove sucrose. The viral pellet was resuspended in 1 mL PBS. The purified virus was adsorbed onto parlodion-coated nickel grids, fixed with 2.5% glutaraldehyde, rinsed with Tris-buffered saline (TBS), and stained with 2% phosphotungstic acid. Images were captured using an FEI Tecnai G2 F20 S-Twin transmission electron microscope (TEM, FEI Co.).

### Real-time PCR

Lung, spleen, and duodenum tissues were homogenized using a Bullet Blender machine. The virus was inoculated into a confluent monolayer of MDBK cells and cultured for 4 days. The inoculated cells were freeze-thawed twice, and the supernatants were collected by centrifugation after 0, 5, and 10 serial passages. Total RNA from these samples was extracted and purified using the RNAiso Plus reagent. The primers used in this study are listed in Supplementary Table S3. First, the copy number of the positive standard was calculated based on the concentration of the positive recombinant plasmid and the copy number formula. Then, 10-fold serial dilutions were performed to generate nine consecutive plasmid DNA gradients of 10^9^ to 10^1^, which were used as templates. Quantitative real-time RT-PCR (qRT-PCR) was performed in 25 μL reactions using a real-time thermocycler and a SYBR Green DNA detection system to amplify target gene sequences and generate standard curves. The copy number of the 5′ UTR gene in each sample was calculated based on the cycle threshold (Ct) value of the standard curve. The housekeeping gene glyceraldehyde-3-phosphate dehydrogenase (GAPDH) was used as an endogenous reference gene.

### Infection of guinea pigs and calves

Three-week-old female SPF Hartley guinea pigs were used for BVDV infection assays. BVDV1 and BVDV3 were administered via nasal inhalation at 100 μL of 10^6^ TCID_50_, respectively. For co-infection, 50 μL of 0.5 × 10^6^ TCID50 BVDV1 and 50 μL of 0.5 × 10^6^ TCID50 BVDV3 were co-administered via nasal inhalation. Guinea pigs were observed daily for clinical signs, body weight, and body temperature for 14 days. Nasal swabs (NS) and whole blood (WB) were collected at 3, 7, 14, 21, and 28 dpi. Surviving guinea pigs were euthanized and necropsied at 28 dpi, and lung, spleen, and duodenum tissues were collected aseptically. Twelve healthy 4-month-old calves, all negative for BVDV (BVDV Total Ab X3 Test, IDEXX), were sourced and maintained at a calf farm in Inner Mongolia, China. The calves were randomly divided into four groups (control, BVDV1, BVDV3, and BVDV1 and BVDV3 co-infection). Each group was administered 3 mL of the respective BVDV strain via nasal inhalation, each containing 3 × 10^6^ TCID50. Calves were monitored for rectal temperature changes for 28 days. Nasal swabs (NS) and whole blood (WB) were collected at 3, 7, 10, 14, 21, and 28 dpi.

### Complete blood count

Blood samples were immediately delivered to the laboratory and analyzed using a hemocytometer with species-specific settings for guinea pig and calf blood (ABX PENTRA80, Sysmex, Japan). The instrument generated a report detailing various blood parameters, including WBC, lymphocytes, and neutrophils.

### Hematoxylin and eosin staining

Lung, spleen, and duodenal tissues were harvested and fixed in 4% buffered neutral formalin for 12 h. A graded series of ethanol concentrations (100, 75, 50, and 25%) was used for dehydration, with each step lasting 30 min, followed by treatment with a 1:1 ethanol and xylene mixture for clearing. The tissue was subsequently treated overnight with a 1:1 paraffin-xylene mixture and embedded in paraffin. Following sectioning into 7 μm slices and staining with hematoxylin and eosin, pathological and morphological changes in the tissues were examined under a ZEISS microscope (ZEISS, HAL100, Gottingen, Germany).

### Virus neutralization tests

Three-week-old female guinea pigs were intramuscularly immunized with 600 μL of a vaccine containing 10^7^ TCID_50_ of inactivated BVDV and an oil-based adjuvant (Montanide ISA 61 VG, SEPPIC) at a 40:60 ratio. Serum samples were collected from the hearts of guinea pigs prior to and 14 days following the initial immunization. The virus neutralization (VN) assay was performed as described by Ståhl et al. (22). In brief, 100 TCID50 of isolated BVDV was mixed with serial two-fold dilutions of the tested sera. Following 1 h of incubation at 37°C, the mixtures were inoculated onto MDBK cells cultured in 96-well plates. After 72 h of incubation at 37°C with 5% CO_2_, an IF assay was conducted on the inoculated MDBK cells. Results were expressed as the reciprocal of the highest serum dilution that inhibited the appearance of specific fluorescence in the inoculated cells.

### Flow cytometry

Apoptosis was assessed via flow cytometry. In brief, MDBK cells and PBMCs were seeded in 6-well plates and incubated for 12 h at 37°C. MDBK cells and PBMCs were infected with BVDV1 and/or BVDV3 at a multiplicity of infection (MOI) of 0.1 and cultured at 37°C for 48 h. MDBK cells were trypsinized and centrifuged at 1000 g for 5 min. The cells were washed twice with cold PBS and then resuspended in 1× binding buffer. Subsequently, 5 μL of annexin V-FITC and 10 μL of propidium iodide were added and incubated for 15 min in the dark (Cat. No. 556420, BD Biosciences). Flow cytometric analysis was performed using a Beckman FACSVerse flow cytometer (CytoFLEX Model A00-1-1102, Beckman). The acquired data were analyzed with FlowJo^™^ v10 Software (BD Biosciences).

### Statistical analysis

Statistical significance was assessed using two-way analysis of variance (ANOVA) or t-tests in GraphPad Prism 7.0 software. Data are presented as the mean ± standard deviation (SD) from at least three independent experiments. Asterisks denote statistically significant differences as follows: ^****^p < 0.0001, ^***^p < 0.001, ^**^p < 0.01, and ^*^p < 0.05.

## Discussion

BRDC encompasses a spectrum of clinical diseases and lesions affecting the respiratory tract of cattle, also referred to as bovine respiratory disease, shipping fever pneumonia, or undifferentiated fever. The etiology of BRDC is multifactorial, involving complex interactions among environmental stressors, host predispositions, and bacterial and viral pathogens (23). Although BVDV is implicated as a contributor to BRDC, acute uncomplicated infections with typical field strains of BVDV rarely result in clinical respiratory disease (13). In China, BVDV1 sub-genotypes are clustered into nine sub-genotypes (1a, 1b, 1c, 1d, 1 m, 1o, 1p, 1q, and 1u), while BVDV2 subtype 2a and BVDV3 subtype 3a have also been detected (24–27). BVDV3 shares genetic and antigenic similarities with BVDV1 and BVDV2. However, studies have shown that fetuses from pregnant cows previously infected with BVDV1 or BVDV2 were not protected against subsequent challenges with BVDV3 (28). Additionally, both modified live virus (MLV) and killed virus (KV) vaccines targeting BVDV1 and BVDV2 generate a comparatively weak cross-reactive antibody response against BVDV3. This reduced efficacy is likely due to antigenic differences between the genotypes, highlighting the need for vaccines specifically designed to target BVDV3 for effective control of its spread (29).

Naturally occurring cases of co-infection with BVDV1 and BVDV3 remain rare, underscoring the need for further exploration into simultaneous infections, post-infection clinical signs, viremia, serological conversion, and blood biochemical tests. BVDV serves as a synergistic agent in mixed respiratory tract infections, inhibiting immune cell function and facilitating easier access for other pathogens to the lungs, resulting in severe disease outcomes (30–32). It is hypothesized that BVDV1 potentiates BVDV3 through immunosuppression (subsequent infection) or synergism (co-infection) (9, 33). In our study, BVDV3 induced apoptosis in cattle cells and guinea pigs, whereas BVDV1 did not. Our findings indicate that co-infection with BVDV1 and BVDV3 leads to severe viremia, reduced WBC counts, and necrocytosis in the lungs of guinea pigs, with two cattle experiencing severe bloody diarrhea. In contrast, single infections with BVDV1 or BVDV3 resulted in minor clinical signs in animals, suggesting that co-infection can lead to more severe disease outcomes compared to single infections.

BVDV-induced MD is a rare but highly fatal manifestation of BVD that occurs in PI cattle harboring NCP BVDV. MD develops when PI cattle are superinfected with CP BVDV, which often originates from a mutation in the resident persistent NCP BVDV (34). The activation of the intrinsic apoptotic pathway is a crucial element in the pathogenesis of MD lesions in cattle persistently infected with BVDV (35). *In vitro* studies using the BL3 lymphoid cell line have demonstrated that a virulent NCP BVDV-2 strain can induce apoptosis, though to a lesser extent than cytopathic BVDV strains. Certain new biotypes of NCP BVDV1 are capable of inducing apoptosis in lymphocytes, which may be associated with immunosuppression (10, 36). Additionally, recent reports have identified virulent BVDV3 strains originating from Brazil, which cause higher morbidity and mortality rates in cattle compared to earlier BVDV3 strains isolated from other regions (2, 37). The BVDV3 strain isolated in this study is closely related to the Brazilian strain, underscoring its potential pathogenicity. In China, BVDV3 strains originating from Brazil have been reported to cause severe respiratory and diarrheal clinical signs, with a high fatality rate in cattle (38). Our BVDV3 strain exhibited strong pathogenicity, capable of inducing significant apoptosis, which may be a major factor in triggering fatal BRDC. Experimental infections with BVDV1 or BVDV2 have been documented in various species, including goats, pigs, rabbits, and guinea pigs, with the infection predominantly manifesting in their upper respiratory tracts. These animals exhibit viral shedding and serological reactions; however, BVDV-like clinical signs are not significant (39–41). Previous experimental infections of cattle, pigs, goats, and sheep with BVDV3 have demonstrated varied clinical signs and serological responses. For instance, pigs infected with BVDV3 exhibited only a low-grade fever without overt clinical signs, and viral RNA was minimally detected in nasal secretions (42). Similarly, goats infected with BVDV3 showed decreased lymphocyte counts and seroconversion, with only a minority exhibiting fever clinical signs. In lambs, severe respiratory signs were observed along with decreased white blood cell (WBC) counts, and viremia appeared at 5 dpi (6, 7).

Because guinea pigs are standard laboratory animals with good experimental reproducibility, are easily susceptible to respiratory diseases, and are frequently used in the establishment of animal virus infection models, they were chosen for this study (43). In our study, guinea pigs were utilized as an experimental model to evaluate BVDV3 infection for the first time. These guinea pigs closely mimicked cattle infections, particularly in light of the rarity of fatal outcomes observed in previous animal models. Notably, co-infection with BVDV1 and BVDV3 resulted in a 40% mortality rate among the guinea pigs. This study documents a fatal outbreak of bovine disease associated with BRDC, identifying NCP BVDV1 and NCP BVDV3 as the primary causal agents. Additionally, the guinea pig infection model emerges as a promising tool for investigating variations in pathogenicity among different isolates and assessing the efficacy of BVDV3 vaccine candidates.

## Data Availability

The original contributions presented in the study are publicly available. This data can be found in the NCBI database: https://www.ncbi.nlm.nih.gov/sra/PRJNA1132692. All data generated or analyzed during this study have been deposited in Figshare with DOI: 10.6084/m9.figshare.26055625.
