# Supplementary material for: Enhanced pathogenicity and synergistic effects of co-infection with bovine viral diarrhea virus 1 and HoBi-like virus in cattle and guinea pigs
Source: Front Vet Sci. 2024 Nov 12;11:1464745. doi: 10.3389/fvets.2024.1464745 (PMC11589818; doi:10.3389/fvets.2024.1464745)
Supplement: Supplementary file 1 [file Table_1.docx]

Supplementary Material

# Supplementary Tables

| Primers | Sequences（5’-3’） |
| --- | --- |
| BVDV1-F | CATGCCCWTAGTAGGACTAGC |
| BVDV1-R | CTCGTCCACRTGGCATCTCGA |
| BVDV3-F | CATACCTTCAGTAGGACGAGC |
| BVDV3-R | TCCATGTGCCATGTACAGCAGAG |
| BRSV-F | TATGCTATGTCCCGATTGG |
| BRSV-R | ACTGATTTGGCTAGTACACCC |

**Table S1.** PCR primers for detecting bovine respiratory pathogens (BVDV1, BVDV3, and BRSV).

| Primers | Sequences（5’-3’） |
| --- | --- |
| BVDV3-5′ UTR-F | CATACCTTCAGTAGGACGAGC |
| BVDV3-5′ UTR-R | TCCATGTGCCATGTACAGCAGAG |
| BVDV3-1F | GCTCTTTGGGAGTACAGCCTGAT |
| BVDV3-1R | CTGCAAGTCACTGCACATTCCT |
| BVDV3-2F | GGTTGGTGCAATTGGTACAACAT |
| BVDV3-2R | CTGTCCATCCATAGGGGCAC |
| BVDV3-3F | CAGGAGGTGTGTGAGGAAACG |
| BVDV3-3R | GCCAGTAACTGCTGATGCAG |
| BVDV3-4F | CTTCACACGTGTGGACTCTGT |
| BVDV3-4R | ATCAGTGATGTGGGGAACCTT |
| BVDV3-5F | GAGAAGTGCCACACTGGAAT |
| BVDV3-5R | AGAACCTGTGGATCTTCCCT |
| BVDV3-6F | TGGCTACAGGGATAACATATGCCT |
| BVDV3-6R | GTGGCATATATGTATGCGGGCA |
| BVDV3-7F | CTCCCAGCAGCAGTGAAGAACAT |
| BVDV3-7R | ACCAGCTCATCAGTAGCTGC |
| BVDV3-8F | GTGGGGATAGCAGTGATGCT |
| BVDV3-8R | GTGGACTAGTTCAATCAGTCTCT |
| BVDV3-9F | GGACAGACCAAACCACGAAGC |
| BVDV3-9R | GTCTTTACCCTTCTTGCTGGT |
| BVDV3-10F | GCATACCAACTAGCACAAGGG |
| BVDV3-10R | CCGGGACCTCAGTCATGTGTT |
| BVDV3-11F | AGTGAGTTTTGACACAAAGGC |
| BVDV3-11R | AATGAGGTAGTTCTCATTAGCCT |
| BVDV1-1F | TCAGCGAAGGCCGAAAAGAGG |
| BVDV1-1R | ACCAGTTGCACCAACCATGCT |
| BVDV1-2F | GTACTGGTGTCCCTTCCCATCT |
| BVDV1-2R | CCATCTGGCCCCTGACTATCTT |
| BVDV1-3F | GACCAGATTGGTGGCCTTATG |
| BVDV1-3R | GAGATATACGCCTTCTCCACT |
| BVDV1-4F | CAACCTTGTGGGCCGACTT |
| BVDV1-4R | GGGCATCCTGAGTCAGTCT |
| BVDV1-5F | TGGTCTGTGACAGCATGGGAC |
| BVDV1-5R | ACCCTCACCCTCTTTTCACAT |
| BVDV1-6F | GTGGAGAGGATCCAGCCAATCT |
| BVDV1-6R | CGACGAATCGCCTCCCTTCT |
| BVDV1-7F | GCGGCAGTTGATTTAGTGGTCT |
| BVDV1-7R | CGTCCTTGAGCCTACCTCCT |
| BVDV1-8F | CAGGTGGAGATCCCTAACTGGT |
| BVDV1-8R | GGCTCATTGAACAAGTCCCATT |
| BVDV1-9F | GCCCGTTGTGATTCCAGGAT |
| BVDV1-9R | AGAGGTCTTCCCTAGTCCAACT |

**Table S2.** PCR primers for amplifying the complete genomes of BVDV3 and BVDV1.

| Primers | Sequences（5’-3’） |
| --- | --- |
| BVDV1-F | CATGCCCWTAGTAGGACTAGC |
| BVDV1-R | CTCGTCCACRTGGCATCTCGA |
| BVDV3-F | CATACCTTCAGTAGGACGAGC |
| BVDV3-R | TCCATGTGCCATGTACAGCAGAG |
| GAPDH -F | AACATCATCCCCGCATC |
| GAPDH -R | CCTCGGTGTAGCCCAAG |
| TNF-α-F | CCTGGTATGAACCCATCTA |
| TNF-α-R | ACCCCAAAGTAGATCTGCC |
| IL-10-F | CAGTTTTATCTAGTGGAGGT |
| IL-10-R | CAGGGAAGAAATCGATGAC |
| IL-15-F | GTGCTTTCTCCTGGAGTT |
| IL-15-R | GCATCCAGATTCTGTTACA |

**Table S3.** qRT-PCR primers for detection of the gene to be tested.
